# Assessing the transcriptional regulation of L-cysteine desulfhydrase 1 in *Arabidopsis thaliana*

**DOI:** 10.3389/fpls.2014.00683

**Published:** 2014-12-04

**Authors:** Ana M. Laureano-Marín, Irene García, Luis C. Romero, Cecilia Gotor

**Affiliations:** Instituto de Bioquímica Vegetal y Fotosíntesis, Consejo Superior de Investigaciones Científicas and Universidad de SevillaSevilla, Spain

**Keywords:** abscission zone, auxin, DES1 promoter, hydathode, floral tissues, promoter-GFP construct

## Abstract

Hydrogen sulfide is an important signaling molecule that functions as a physiological gasotransmitter of comparable importance to NO and CO in mammalian systems. In plants, numerous studies have shown that sulfide increases tolerance/resistance to stress conditions and regulates essential processes. The endogenous production of hydrogen sulfide in the cytosol of *Arabidopsis thaliana* occurs by the enzymatic desulfuration of L-cysteine, which is catalyzed by the L-cysteine desulfhydrase enzyme DES1. To define the functional role of DES1 and the role that the sulfide molecule may play in the regulation of physiological processes in plants, we studied the localization of the expression of this gene at the tissue level. Transcriptional data reveal that *DES1* is expressed at all developmental stages and is more abundant at the seedling stage and in mature plants. At the tissue level, we analyzed the expression of a GFP reporter gene fused to promoter of *DES1*. The GFP fluorescent signal was detected in the cytosol of both epidermal and mesophyll cells, including the guard cells. GFP fluorescence was highly abundant around the hydathode pores and inside the trichomes. In mature plants, fluorescence was detected in floral tissues; a strong GFP signal was detected in sepals, petals, and pistils. When siliques were examined, the highest GFP fluorescence was observed at the bases of the siliques and the seeds. The location of GFP expression, together with the identification of regulatory elements within the *DES1* promoter, suggests that DES1 is hormonally regulated. An increase in *DES1* expression in response to ABA was recently demonstrated; in the present work, we observe that *in vitro* auxin treatment significantly repressed the expression of DES1.

## Introduction

Hydrogen sulfide, a known toxic molecule, is considered to be an important signaling molecule. In animal systems, hydrogen sulfide functions as physiological gasotransmitter; this molecule is recognized to be of equal importance to NO and CO and has been the subject of many reviews (Gadalla and Snyder, 2010; Kimura, 2011; Wang, 2012). H_2_S is mostly catalyzed via the enzymatic reactions of cystathionine β-synthase (CBS) and cystathionine γ-lyase (CSE) (Wang, 2012) in mammals. Both enzymes are known for their participation in the transsulfuration pathway, which is critical for the synthesis of cysteine from methionine. Both CBS and CSE use pyridoxal 5′-phosphate as a cofactor and are exclusively located in the cytosol (Gadalla and Snyder, 2010; Wang, 2012).

In recent years, hydrogen sulfide has been also shown to be a signaling molecule in plants similar to NO and H_2_O_2_. Numerous studies have demonstrated the role of sulfide in protection against numerous stress conditions. Additional studies have demonstrated that this molecule is involved in regulating essential processes such as photosynthesis, stomatal movement, senescence, and autophagy. Consequently, several reviews in plant systems have been recently released (Garcia-Mata and Lamattina, 2013; Lisjak et al., 2013; Calderwood and Kopriva, 2014; Gotor et al., 2014; Hancock and Whiteman, 2014).

Hydrogen sulfide is biosynthesized in plant chloroplasts during the photosynthetic sulfate assimilatory process by the sulfite reductase that reduces sulfite to sulfide. Due to the high toxicity of hydrogen sulfide, it is rapidly incorporated into carbon skeletons to form cysteine by the O-acetylserine(thiol)lyase (OASTL) enzymes. OASTL enzymes are found in the cytosol, plastids and mitochondria and are encoded in *Arabidopsis thaliana* by the *OAS-A1, OAS-B*, and *OAS-C* genes, respectively (Takahashi et al., 2011; Romero et al., 2014). In mitochondria, H_2_S is also produced during the detoxification of cyanide by β-cyanoalanine synthase; this enzyme catalyzes the conversion of cysteine and cyanide to hydrogen sulfide and β-cyanoalanine. Like cyanide, sulfide is a potent inhibitor of mitochondrial cytochrome c oxidase. Sulfide in the mitochondria must be detoxified by OAS-C to produce cysteine, thus generating a cyclic pathway for cyanide/sulfide detoxification (Garcia et al., 2010; Alvarez et al., 2012b).

H_2_S is also produced in plants by cysteine-degrading enzymes, such as D- and L-cysteine desulfhydrases; these enzymes also produce pyruvate and ammonium (Riemenschneider et al., 2005; Alvarez et al., 2010). We have recently shown that the protein DES1 is a pyridoxal-5′-phosphate-dependent L-cysteine desulfhydrase located in the cytosol of *Arabidopsis* (Alvarez et al., 2010). Therefore, the H_2_S levels in the cytosol are determined via the coordinated enzymatic activities of OAS-A1 and DES1 (Gotor et al., 2014; Romero et al., 2014).

Hydrogen sulfide is weakly acidic and dissociates in aqueous solutions into H^+^ and HS^−^. In this ionized form, hydrogen sulfide cannot permeate membranes (Kabil and Banerjee, 2010). In the basic pH of the chloroplast stroma under illumination, and in the mitochondrial stroma in metabolically active cells, sulfide is predominantly found in the charged HS^−^ form. Therefore, hydrogen sulfide is unable to cross out the chloroplast and mitochondrial membranes. Accordingly, DES1 is the responsible for the production of sulfide in the plant cytosol (Romero et al., 2013), with an estimated steady-state concentration of 50 μM (Krueger et al., 2009).

Recent studies have concluded that DES1 modulates the generation of sulfide for signaling in important plant processes, such as the progression of autophagy and the stomatal movement. Irrespective of nutrient conditions, it was demonstrated that sulfide exerts a general effect on autophagy in plants through negative regulation of this process (Alvarez et al., 2012a; Gotor et al., 2013). It has been recently demonstrated that sulfide generated by DES1 acts upstream of nitric oxide in the ABA signaling network in stomatal guard cells (Scuffi et al., 2014).

To gain insight into the regulation of DES1, we analyzed the tissue and cellular localization of DES1 using a *DES1* promoter-GFP construct. We found maximum levels of gene expression in the seedling and mature stages of plant development. We were able to further localize the GFP signal to vegetative and reproductive tissues in correlation with the hormonal regulation of DES1.

## Materials and methods

### Plant material, growth conditions and treatments

*Arabidopsis thaliana* wild type ecotype Col-0 and the transgenic *PromDES1-GFP* line were used in this work. Plants were grown in soil for 6 weeks with a photoperiod of 16 h of white light (120 μE m^−2^ s^−1^) at 20°C and 8 h of dark at 18°C. Alternatively, surface sterilized seeds were germinated and grown in agar-supplemented Murashige and Skoog (MS) medium for 1–2 weeks. For the auxin treatments, wild type Col-0 seeds were germinated and grown for 7 days on MS plates in the presence of 0.1 or 1 μM of indoleacetic acid (IAA).

### DNA cloning and plasmid construction

To clone the *DES1* promoter, a 3 kb of the genomic sequence upstream from the *DES1* gene start codon was amplified using specific primers. Total DNA was isolated from young *Arabidopsis* leaves using the Qiagen DNeasy Plant Minikit. The 3 kb sequence containing the *DES1* promoter was amplified by PCR using the primers proDES1-F: CACCCATTTTATTTTACACCACG and proDES1-R: GTGGTTTGTCTTTGGAAAACT and the Invitrogen proofreading Platinum Pfx DNA polymerase. PCR conditions were as follows: a denaturation cycle of 2 min at 94°C, followed by 35 amplification cycles of 15 s at 94°C, 30 s at 55°C, and 1 min at 68°C. The amplified region was then ligated into the Invitrogen pENTR/D-TOPO vector using the Invitrogen Directional TOPO Cloning Kit following the manufacturer's instructions. Positive clones were identified by PCR and chosen for plasmid DNA isolation. Using Invitrogen Gateway® technology, the *DES1* promoter was then cloned into the pMDC110 vector (Curtis and Grossniklaus, 2003), a plant expression vector for the construction of promoter-reporter GFP vectors. The final construct for used for plant transformation was identified by colony PCR and plasmid PCR. The construction was named *PromDES1-GFP*.

### Transformation of *Arabidopsis*

For plant transformation, the construct *PromDES1-GFP* was transformed into an *Agrobacterium tumefaciens* strain and then introduced into *A. thaliana* plants by dipping the developing floral tissues into a solution containing the *A. tumefaciens* strain, 5% sucrose, and 0.005% (v/v) of the surfactant Silwet L-77 (Clough and Bent, 1998). Transgenic plants were recovered by selecting seeds on solid MS medium containing 50 mg/l of hygromycin.

### Real-time RT-PCR

Quantitative real-time RT-PCR was used to analyze the expression of *DES1* and *OAS-A1* genes. Total RNA was extracted from different tissues of *Arabidopsis* plants or the aerial parts of *Arabidopsis* seedlings using the Qiagen RNeasy Plant Mini Kit. RNA was reverse transcribed using an oligo(dT) primer and the Invitrogen SuperScript First-Strand Synthesis System for RT-PCR following manufacturer's instructions. Gene-specific primers for each gene were designed using the Invitrogen Vector NTI Advance 10 software. Primer sequences were as follows: qDES1-F, 5′-TCGAGTCAGTCAGATATGAAGCT-3′ and qDES1-R, 5′-TGTAACCTTGGTACCAACATCTCT-3′ for the *DES1* gene; qOASA-F, 5′-CACGAGCGATTTTCTCCATT-3′ and qOASA-R, 5′-CAATTCTCGAGGCCATGATT-3′ for the *OAS-A1* gene; qUBQ-F, 5′-GGCCTTGTATAATCCCTGATGAATAAG-3′ and qUBQ-R, 5′-AAAGAGATAACAGGAACGGAAACATAGT-3′ for the constitutive *UBQ10* gene. The PCR efficiency of all primer pairs was determined to be close to 100%. Real-time PCR was performed using the Bio-Rad iQ SYBR Green Supermix. Signals were detected on a Bio-Rad iCYCLER according to the manufacturer's instructions. The cycling profile consisted of 95°C for 10 min followed by 45 cycles of 95°C for 15 s and 60°C for 1 min. A melting curve from 60°C to 90°C was run following the PCR cycling. The expression levels of the genes of interest were normalized to that of the constitutive *UBQ10* gene by subtracting the cycle threshold (CT) value of *UBQ10* from the CT value of the gene (ΔCT). The results shown are means ± SD of at least three independent RNA samples.

### GFP localization by confocal microscopy

Tissues from *Arabidopsis* at different developmental stages were visualized using a Leica TCS SP2 spectral confocal microscope. Samples were excited using the 488 nm line of an argon ion laser; emission was detected between 510 and 580 nm for GFP imaging (pseudocolored green) and between 620 and 680 nm for chloroplast autofluorescence (pseudocolored red). The microscopy images were processed using the Leica Confocal Software.

## Results

### Isolation of the *DES1* promoter region and production of promoter-reporter transgenic plants

Recent work has suggested that DES1 modulates the generation of sulfide in the cytosol for signaling purposes (Gotor et al., 2013; Romero et al., 2013). Mutations in *DES1* result in premature leaf senescence in mature plants, which can be observed at transcriptional and cellular levels; and at the seedling stage, an increased tolerance to abiotic stress is observed (Alvarez et al., 2010, 2012a). To determine the role of DES1 in plant growth and development, we examined the spatial and temporal regulation of *DES1* gene expression. For this purpose, promoter-GFP transgenic plants were constructed using a 3002 bp fragment isolated from the *DES1* promoter region. This fragment comprises the genomic region upstream from the *DES1* gene and its first intron. The intron was included based on a previous report demonstrating that the first intronic region of the *OAS-A1* gene, other member of the OASTL family, includes essential elements for tissue-specific expression (Gutierrez-Alcala et al., 2005). Thus, the *DES1* promoter consists of 2836 bp from the intergenic region between *DES1* (At5g28030) and the upstream gene At5g28040, 14 bp of the first exon containing the 5′-UTR region, 118 bp of the first intron and 34 bp of the second exon that contains the remainder of the 5′-UTR region, immediately upstream of the translation initiation site (Supplemental Figure 1; www.arabidopsis.org). The promoter sequence was analyzed for cis-acting regulatory elements using available web tools (AthaMapMan; AGRIS; PLACE). Several binding site motifs were detected, including ABA- and Auxin-related elements and leaf development and senescence-regulatory elements (Table 1).

**Table 1:** **List of various cis-regulatory elements and their positions in the *DES1* promoter**.

	**Function**	***cis* element**	**Sequence**	**Position**	**TF family**
1	Defense against insect herbivory	AtMYC2 BS in RD22	CACATG	(1578-1583)	BHLH
2	Development	Bellringer/Replumless /Pennywise	AAATTAAA	(2593-2600)	Homeobox
3	Development	Bellringer/Replumless /Pennywise	AAATTAGT	(1368-1375)	Homeobox
4	Development	Bellringer/Replumless /Pennywise	ACTAATTT	(293-300)	Homeobox
5	Response to hiperosmolarity	ATB2/AtbZIP53/AtbZlP44/GBF5 BS in ProDH	ACTCAT	(2537-2542)	bZIP
6	Auxins response	ARFI binding site motif	TGTCTC	(177-182) (1820-1825)	ARF
7	ABA response	DPBF1&2 binding site motif	ACACTAG	(897-904)	bZIP
8		MYB binding site promoter	AACCAAAC	(2359–2366)	MYB
9	Defense and stress	MYB4 binding site motif	AACAAAC	(825-831) (775-781) (2446-2452)	MYB4
10	Leaf maturation and senescence	RAVI-A binding site motif	CAACA	(1-5) (2138-2142) (1508-1512) (583-587) (360-367)	ABI3VPl
11	Development	LFY consensus binding site motif	CCAATG	(1413-1418)	LFY
12		Boxll promoter motif	GGTTAA	(2810-2815)	
13	Drought response	DRE-like promoter motif	TACCGACCA	(533-541)	
14	Light response	GATA promoter motif [LRE]	TGATAG	(2957-2962)	
15	Light response	GATA promoter motif [LRE]	AGATAA	(287-292) (96-101)	
16	Light response	GATA promoter motif [LRE]	TGATAA	(2308-2313)	
17		Hexamer promoter motif	CCGTCG	(649-654)	
18		T-box promoter motif	ACTTIG	(1529-1534)	

*The immediate upstream nucleotide of the start codon is designated as position 1 as shown in the Supplemental Figure 1*.

The *DES1* promoter was fused to the *GFP* gene. The plant transformation construct was named *PromDES1-GFP*. Six transgenic *A. thaliana* plants were obtained; and homozygous lines were analyzed by laser confocal microscopy for *in vivo* GFP detection. One T4 line was selected for further studies.

### Developmental *DES1* expression profiles in *Arabidopsis* wild type plants

**Figure 1:**
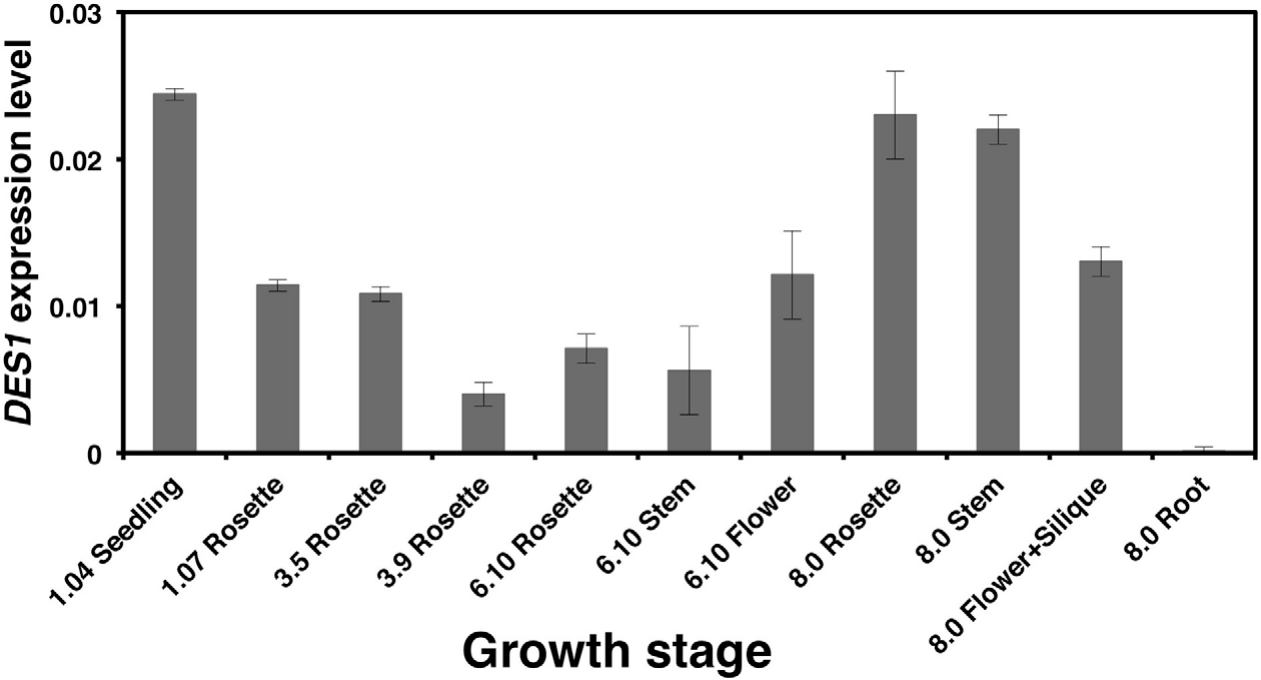
**Relative expression levels of the *DES1* gene in different tissues at different growth stages**. Wild type *Arabidopsis* plants were grown in soil under the growth conditions described in Materials and Methods. At the indicated growth stages, the tissues were collected for real-time RT-PCR analysis. For the growth stage 1.04, seeds were grown on solid MS medium without sucrose in Petri dishes. The *DES1* transcript levels were normalized to an internal control, the constitutively expressed *UBQ10* gene. Data shown are mean values ± SD from three independent analyses.

To investigate the transcriptional regulation of the *DES1* gene, we first examined its expression profile during the development and in different tissues of wild type *Arabidopsis* plants, using real-time RT-PCR analysis. Tissues were harvested either from seedlings grown on MS plates without sucrose or from plants grown in soil at different growth stages up to maturity (Boyes et al., 2001). The highest *DES1* expression levels were detected in leaf tissues at the beginning and end of plant development; this corresponded to 14-day-old seedlings (growth stage 1.04) and to 35-day-old plants (growth stage 8.0). Flowering was completed at growth stage 8.0, at least under our experimental conditions (Figure 1). The lowest *DES1* expression level found in leaves was observed at growth stage 3.9, which corresponded to plants where rosette growth was complete but before flower buds were visible. Curiously, the *DES1* expression levels in reproductive tissues (flowers and siliques) were significantly greater compared to rosette leaves in plants at vegetative growth stages.

### GFP expression driven by the *DES1* promoter in vegetative tissues

**Figure 2:**
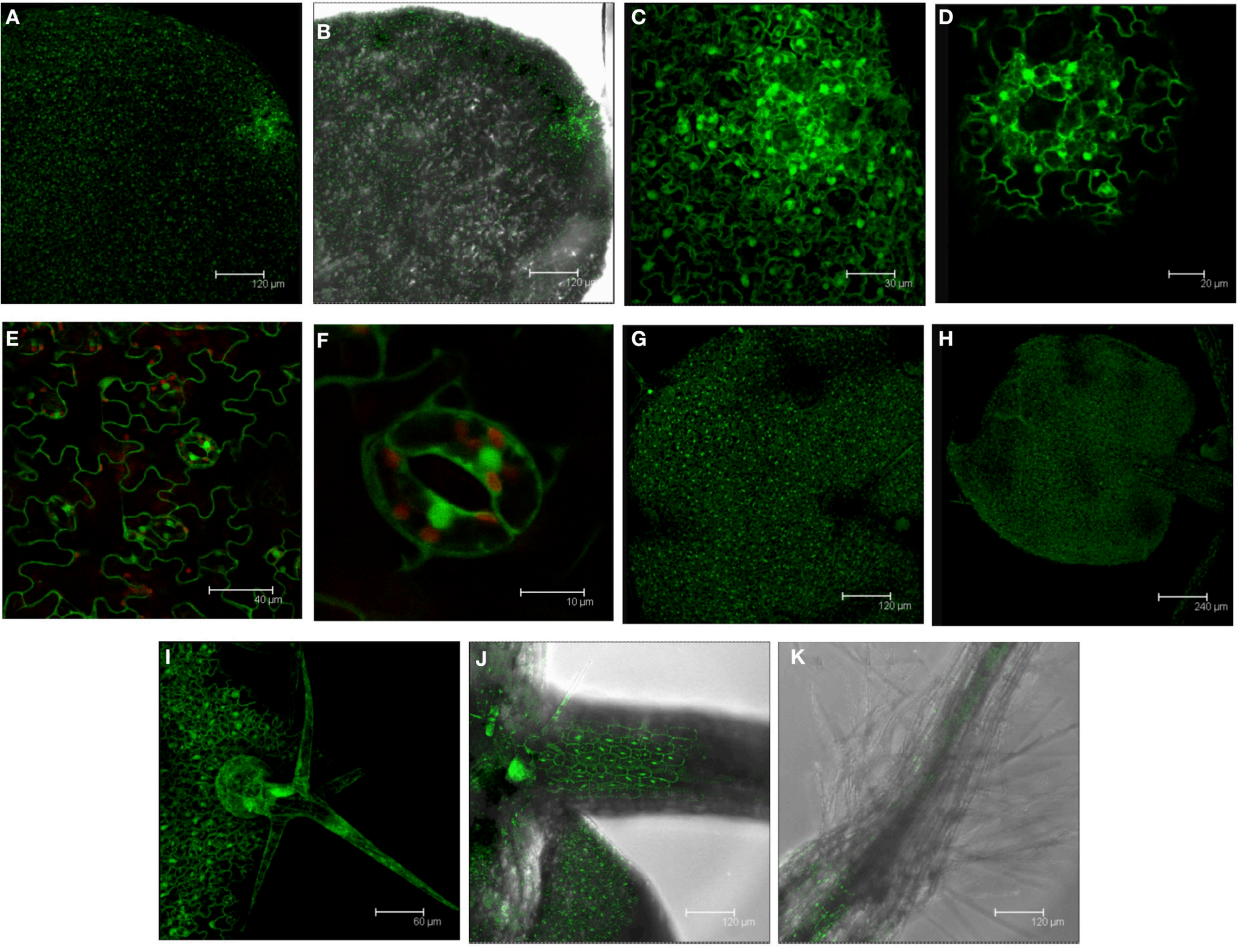
**GFP localization in 7-day-old seedlings from the *PromDES1-GFP* transgenic line**. Transgenic *Arabidopsis PromDES1-GFP* plants were grown on solid MS plates for 7 days. GFP was visualized using confocal fluorescence microscopy. **(A,B)** GFP image and the same image with overlapping transmitted light image from the abaxial side of a leaf. **(C)** Magnification of **(A)** showing a hydathode pore. **(D)** GFP image of a hydathode pore in a different leaf. **(E)** GFP image with overlapping chloroplast autofluorescence image of epidermal cells on the adaxial side of a leaf. **(F)** Magnification of **(E)** showing a stoma. **(G,H)** GFP images from the adaxial side of two different leaves. **(I)** GFP image of a trichome. **(J)** GFP image with overlapping transmitted light image of a petiole. **(K)** GFP image with overlapping transmitted light image of a root neck. All images shown are Z-stacks of optical sections.

The tissue-specific expression of the *DES1* gene was examined further using the promoter-GFP approach. GFP was visualized in *PromDES1-GFP* plants using confocal microscopy. GFP expression largely correlated with *DES1* expression profiles in wild type plants at the whole tissue level. At the seedling stage, we detected GFP fluorescence in the whole leaf; fluorescence was observed initially at 7 days after sowing (Figure 2). A closer examination of the abaxial side of the leaf revealed some specific sites with high GFP accumulation. High expression at the very tip of the leaf would correspond with hydathode pores (Figures 2A–D). In epidermal cells, we localized the GFP signal to the thin layer of cytoplasm underneath the cell wall; this included the guard cells of the stomata (Figures 2E,F). The localization of GFP to the nucleus that we observed was likely due to the relatively small size GFP, which can translocate to the nucleus on its own through nuclear pores (Seibel et al., 2007).

GFP localization was also observed throughout the adaxial side of the leaf in 7-day-old seedlings. Obscure zones with no fluorescence corresponded to trichomes growing upwards in the vertical plane (Figures 2G,H). A strong GFP signal was observed inside the trichomes and in the trichome basement cells; GFP was localized to the cytoplasmic strands and clearly detectable (Figure 2I). GFP also appeared in the base of the petiole (Figure 2J). In root tissues, GFP was only observable in the hypocotyl-root transition zone (Figure 2K).

**Figure 3:**
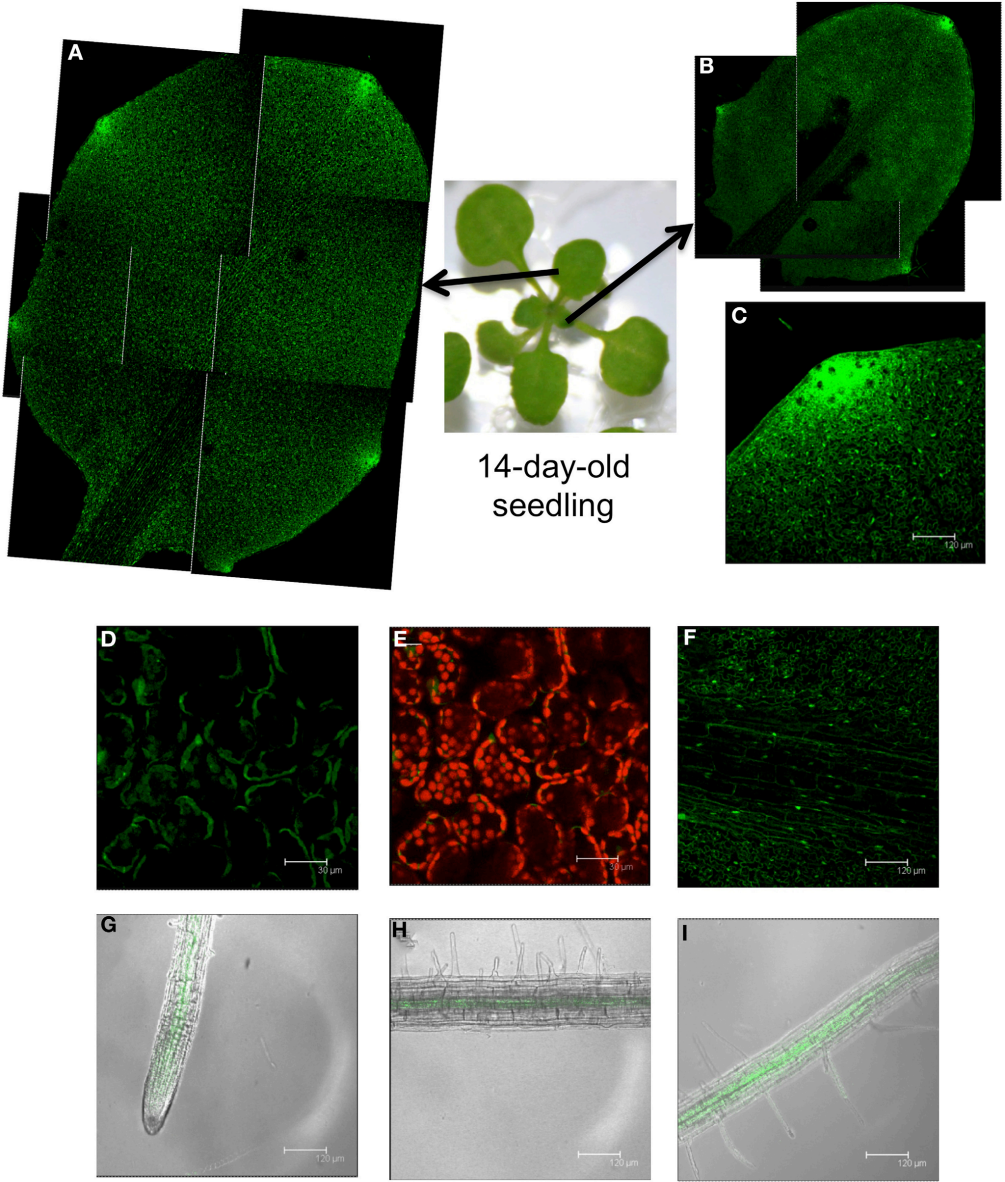
**GFP localization in 14-day-old seedlings from the *PromDES1-GFP* transgenic line**. Transgenic *Arabidopsis PromDES1-GFP* plants were grown on solid MS plates for 14 days. GFP was visualized using confocal fluorescence microscopy. **(A,B)** Reconstruction of two different leaves from a 14-day-old seedling by joining the GFP images from different sections. **(C)** GFP image of a hydathode pore. **(D,E)** GFP image and the same image with overlapping red chloroplast autofluorescence of mesophyll cells. **(F)** GFP image of a leaf showing the vascular tissue. **(G–I)** GFP image with overlapping transmitted light image from different root sections. All images shown are Z-stacks of optical sections.

At the 1.04 growth stage (14-day-old seedlings), the GFP signal increased and was distributed throughout the leaf (Figure 3). Accordingly, the maximum GFP localization was associated with the hydathode pores; the larger the leaf, the greater number of hydathodes contained (Figures 3A–C). A closer look at the mesophyll cell layer (Figures 3D,E) and leaf vascular tissues (Figure 3F) also revealed GFP expression. At this growth stage, GFP was detectable in root tissues; fluorescence was mostly observed in the meristematic zone and vascular tissues (Figures 3G–I).

### GFP expression driven by the *DES1* promoter in reproductive tissues

**Figure 4:**
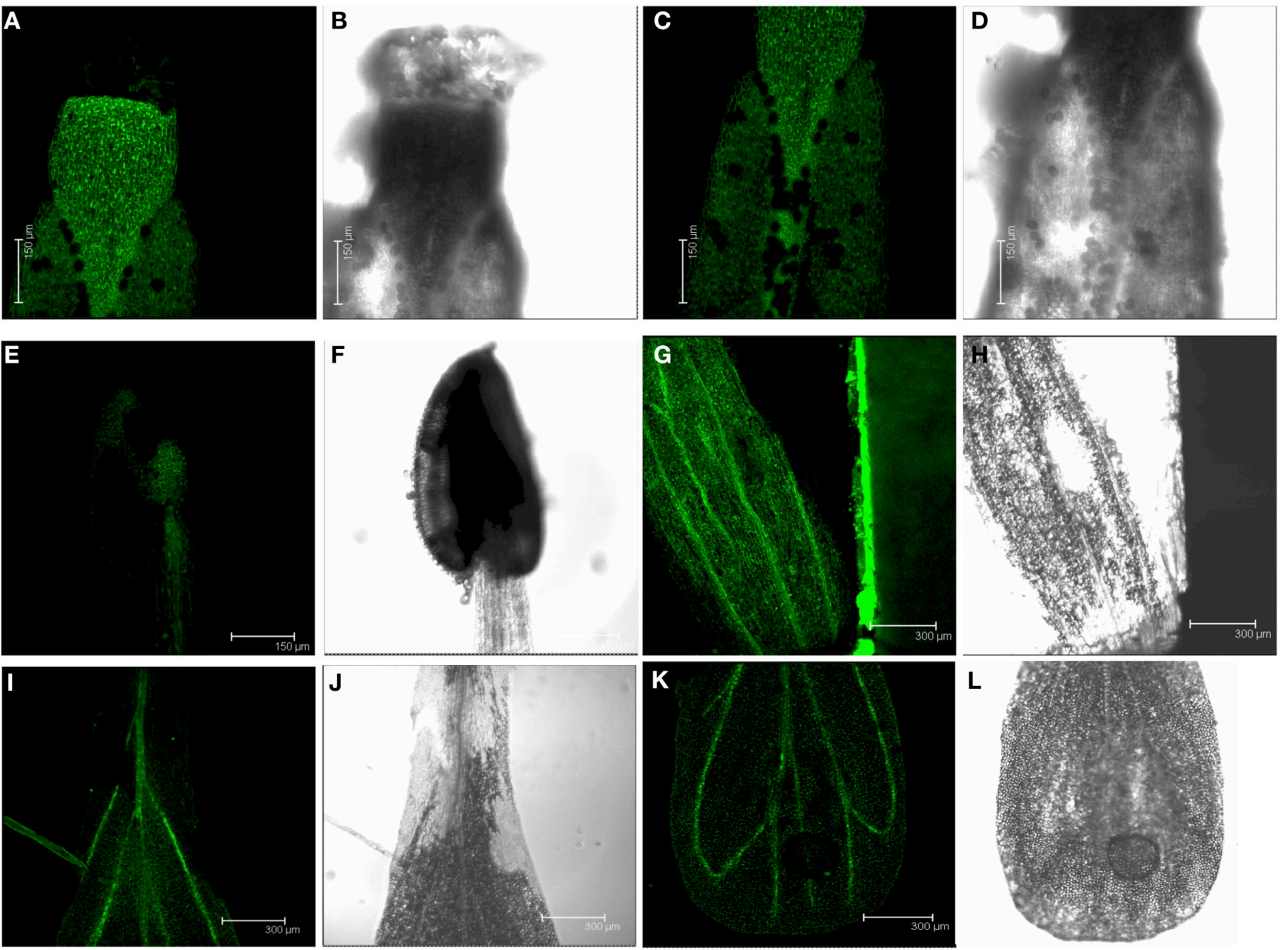
**GFP localization in the reproductive tissues of plants from the *PromDES1-GFP* transgenic line**. Transgenic *Arabidopsis PromDES1-GFP* plants were grown in soil under physiological growth conditions for 35–40 days. GFP was visualized using confocal fluorescence microscopy. **(A,B)** GFP and transmitted light images of the upper part of a pistil. **(C,D)** GFP and transmitted light images of the base of a pistil. **(E,F)** GFP and transmitted light images of a stamen. **(G,H)** GFP and transmitted light images of a sepal. **(I–L)** GFP and transmitted light images of whole petal. All images shown are Z-stacks of optical sections.

At the mature stage, the promoter-GFP approach also confirmed previous data concerning *DES1* expression at the organ level. A significant level of GFP fluorescence was observed in floral tissues (Figure 4). In open flowers, a strong GFP signal was detected in the upper pistil and at the base of the pistil; only a very weak signal was observed in the stigma. The ovules inside the pistil were clearly distinguishable as black dots against the green GFP signal; this indicated that no GFP expression occurred in these cells (Figures 4A–D). A lower level of GFP fluorescence was observed in the stamen and was detectable both in the anther and the filament (Figures 4E,F). The GFP signal in the sepals and petals of the flower was high in the vascular tissues; GFP expression appeared to be significantly greater in the sepal than in the petal (Figures 4G–L).

**Figure 5:**
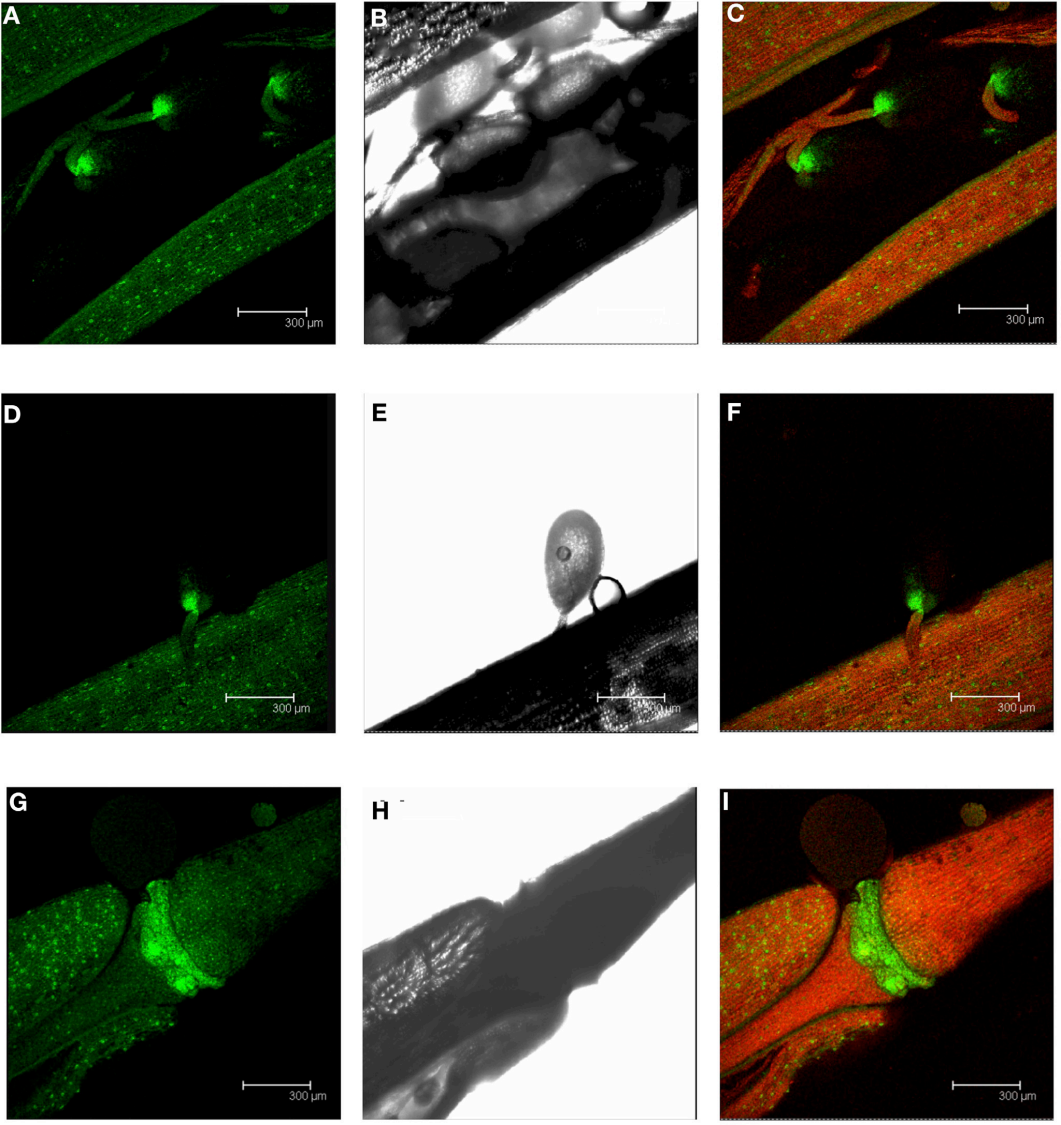
**GFP localization in developing siliques of plants from the *PromDES1-GFP* transgenic line**. Transgenic *Arabidopsis PromDES1-GFP* plants were grown in soil under physiological growth conditions for 40 days. GFP was visualized using confocal fluorescence microscopy. **(A–C)** GFP image, transmitted light image, the same GFP image with overlapping transmitted light and red chloroplast autofluorescence image of a developing silique containing several immature seeds. **(D–F)** GFP image, transmitted light image, the same GFP image with overlapping transmitted light and red chloroplast autofluorescence image of an immature seed. **(G–I)** GFP image, transmitted light image, the same GFP image with overlapping transmitted light and red chloroplast autofluorescence image in the abscission zone of a silique. All images shown are Z-stacks of optical sections.

We also examined the green siliques containing developing seeds. A GFP signal was detected in the valve of the silique and the highest GFP expression was observed at the base of the seeds (Figures 5A–C). A closer look at the seed showed that the high GFP fluorescence appeared to be associated with the seed abscission zone (Figures 5D–F). An intense GFP signal was also found at the base of the siliques, which likely corresponds to the silique abscission zone (Figures 5G–I).

### Regulation of *DES1* expression by exogenous auxins

**Figure 6:**
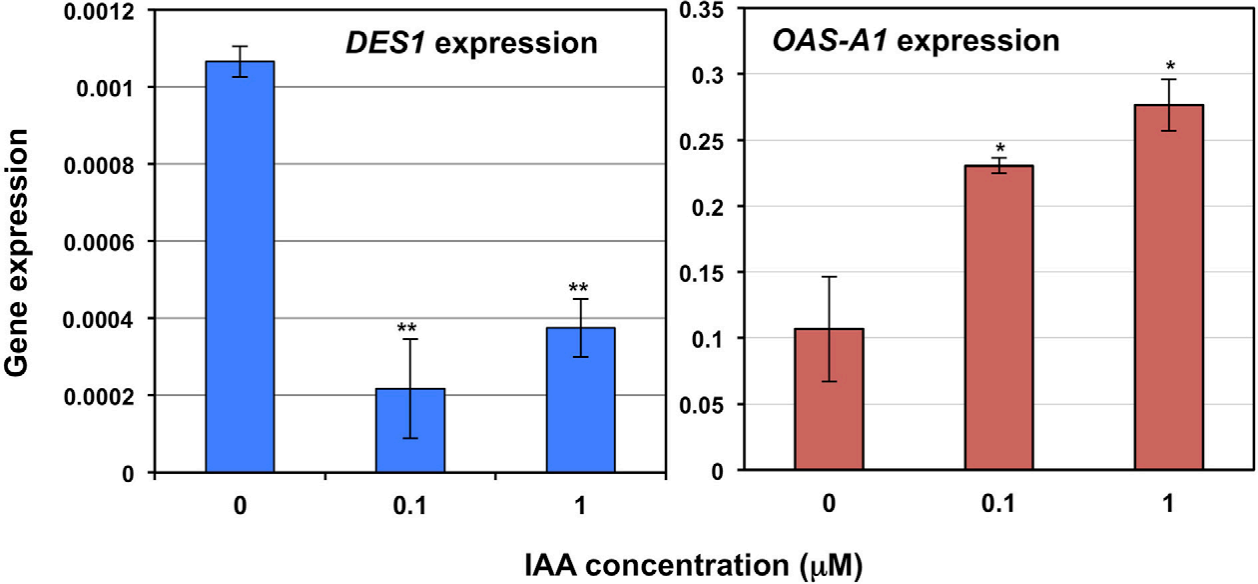
**The effect of exogenous auxins on the expression levels of *DES1* and *OAS-A1* genes in wild type plants**. Wild type *Arabidopsis* plants were grown on solid MS plates for 7 days in either the absence or presence of IAA at the indicated concentrations. Whole seedlings were then collected for real-time RT-PCR analysis. The *DES1* and *OAS-A1* transcript levels were normalized to the internal control, the constitutively expressed *UBQ10* gene. Data shown are mean values ± SD from three independent analyses. The one-factor analysis of variance (ANOVA) statistical analysis of the data was performed using the program OriginPro 7.5. ^**^P < 0.01; ^*^P < 0.05.

The spatial distribution of GFP expression conferred by the *DES1* promoter suggests that DES1 is regulated by the hormone auxin (Teale et al., 2006; Wang et al., 2011; Basu et al., 2013; Baylis et al., 2013). Therefore, we analyzed *DES1* gene expression in response to the exogenous application of auxins. Seeds were germinated directly on indole-3-acetic acid (IAA) at two different concentrations. After 7 days of growth, the level of *DES1* gene expression was determined and compared to the level of gene expression in plants grown in the absence of IAA (Figure 6). We observed a strong and significant reduction in the level of *DES1* expression in the presence of the auxin at a lower concentration of 0.1 μM. In the same samples, we measured the level of *OAS-A1* gene expression. OAS-A1 is the cytosolic enzyme that acts in an opposite manner to DES1. A strong and significant induction in the expression level of *OAS-A1* was observed (Figure 6).

## Discussion

Recent investigations have changed the view of the *A. thaliana* L-Cys desulfhydrase 1 (DES1) protein from a minor and auxiliary enzyme belonging to the OASTL protein family to an important and essential enzyme that regulates the homeostasis of cysteine and modulates the generation of sulfide in the cytosol for signaling purposes (Gotor et al., 2014; Romero et al., 2014). Consequently, knowledge of the tissue-specific localization and regulation of the enzyme will help us to understand the mechanisms underlying the specific functions of DES1.

At the protein level, DES1 has very low abundance; this was confirmed by the identificaction of a small number of peptides in proteomic analysis (AtProteome Database). The steady-state *DES1* transcript levels are also substantially low. For example, the expression level of the *DES1* gene is approximately two orders of magnitude lower that the *OAS-A1* expression level; this is illustrated in Figure 6, and easily verifiable using available web resources (www.arabidopsis.org; www.genevestigator.com). When GFP fluorescence is observed using the promoter-GFP approach, gene expression driven by the *DES1* promoter is relatively high. Fluorescence is mainly observed throughout the whole leaf in early growth stages and in reproductive tissues. These results suggest that DES1 is regulated at post-transcriptional or post-translational level. Such a hypothesis makes sense considering the function of this protein in the generation of sulfide in the cytosol to be used for signaling in important processes such as autophagy (Alvarez et al., 2012a; Gotor et al., 2013; Romero et al., 2013). Sulfide is a toxic molecule; in recent years, it has been further recognized as an important signaling molecule in animal and plant systems (Gadalla and Snyder, 2010; Kimura, 2011; Wang, 2012; Garcia-Mata and Lamattina, 2013; Lisjak et al., 2013; Calderwood and Kopriva, 2014; Hancock and Whiteman, 2014). Therefore, sulfide generation activity in the cytosol should be precisely regulated to avoid deleterious effects. Further investigation will be necessary to determine timing, tissue regulation and the players responsible of this sulfide tuning. Sulfide has been implicated in the regulation of other essential process such as stomatal movement (Garcia-Mata and Lamattina, 2010; Lisjak et al., 2010). Very recently, the involvement of DES1 in the ABA-dependent signaling network in guard cells and the requirement for DES1 in ABA-dependent NO production have been demonstrated (Scuffi et al., 2014). These data suggest that the DES1 protein may be hormonally regulated and may crosstalk with other signaling molecules.

The present study demonstrates that the maximum *DES1* expression occurs at the initial (seedling) and final (maturity) stages of plant development. This suggests a specific role for DES1 at these developmental stages. These data fit well with data gathered using *des1* null mutants, in which phenotypic differences were observed at these stages. ROS production was practically unchanged after cadmium treatment in *des1* mutant seedlings, in contrast with wild type seedlings. Consequently, the *DES1* mutation produces an enhanced tolerance to cadmium and H_2_O_2_ stress conditions (Alvarez et al., 2010). At maturity, mutation in the *DES1* gene leads to premature leaf senescence and promotes the accumulation and lipidation of the ATG8 protein; ATG8 is typically associated with the induction of autophagy. The transcriptional profile of the *des1* mutant corresponds with the observed premature senescence and induced autophagy phenotypes. Most important, the *DES1* mutation significantly alters the transcriptional profile at the late growth stage. When transcriptomic analysis was performed using leaves from plants grown for 20 d (growth stage 3.9), only 16 genes in the *des1* mutant were differentially expressed compared to wild type plants. In contrast, the *des1* transcriptional profile changed dramatically compared to wild type in leaves from plants grown for 30 d (growth stage 6.3). The normalized data revealed that 1614 genes were differentially expressed in the mutant compared to the wild type (Alvarez et al., 2012a). Consequently, the function of DES1 seems to be critical at this late growth stage.

An examination of GFP expression driven by the *DES1* promoter in vegetative tissues reveals that the highest GFP signal occurs in the hydathode pores distributed along the margin of the leaf; the number of pores increases with the leaf size. Hydathodes are specialized pore-like structures that act as the exit point in vascular tissues. At these sites, water and ions are released from the xylem. It has also suggested that hydathodes are involved in ion reabsorption to other tissues through the phloem (Nagai et al., 2013). In addition, the hydathodes are open pores similar to stomata (Nagai et al., 2013). The *Arabidopsis* basic helix-loop-helix (bHLH) protein MUTE, which is a master regulator of stomatal differentiation, is also required for the production of hydathodes (Pillitteri et al., 2008). We have detected a significant GFP fluorescence signal localized to the cytoplasm of guard cells, which suggests the DES1 protein or the sulfide generated by DES1 has a specific function in these pore structures in *Arabidopsis* leaves. This suggestion is reinforced by our recent findings that show DES1 is required for ABA-dependent stomatal closure and the sulfide generated by DES1 acts upstream of nitric oxide in this signaling network (Scuffi et al., 2014).

The *DES1* promoter also confers strong GFP expression inside the trichomes; this result supports numerous reports that have demonstrated the significance of this cell type in relation to sulfur metabolism. *In situ* hybridization studies in combination with determinations of glutathione content by confocal microscopy demonstrated that highly active glutathione biosynthesis occurs in *Arabidopsis* trichome cells (Gotor et al., 1997; Gutierrez-Alcala et al., 2000). Furthermore, protein profiling performed in this specific cell type also identified an important number of proteins involved in sulfur metabolism (Wienkoop et al., 2004). This trichome-specific expression driven by the *DES1* promoter is similar to expression driven by the *OAS-A1* promoter (Gutierrez-Alcala et al., 2005). These findings suggest that, in this cell type, the homeostasis of cysteine is important and is modulated by the enzymes OAS-A1 and DES1; OAS-A1 catalyzes the synthesis of cysteine and DES1 catalyzes the degradation of cysteine.

In this work, a detailed analysis of the expression of a reporter gene driven by the promoter of a gene encoding an enzyme involved in plant sulfur assimilation was performed for the first time. The localization of reporter expression conferred by such promoters in reproductive tissues was previously unknown. In open flowers, the *DES1* promoter confers high GFP expression, which occurs mainly in the pistil, sepal, and petal. Weaker GFP signals were observed in the stamen. The presence of *OAS-A1* transcripts was also detected in flowers by *in situ* hybridizations; this finding was analogous to our observations in trichomes (Gotor et al., 1997).

When siliques were analyzed, strong GFP fluorescence was detected in the presumed abscission zones at the bases of the siliques and seeds. Cell separation is a process highly regulated by plant hormones. Ethylene, JA, and ABA act together to regulate organ abscission (Ogawa et al., 2009). Auxin is also involved in many abscission events (Basu et al., 2013).

In general, the tissue-specific expression pattern of GFP conferred by the *DES1* promoter supports the hormonal regulation of DES1. We have identified cis-elements located within the promoter sequence that correlate with specific hormonal regulation; these include ABA response elements (DPBF binding site motifs), drought response elements (DRE-like promoter motifs), and auxin response elements (ARF1 binding site motifs), and several stress-responsive binding site motifs. The *DES1* promoter also contains a number of light responsive elements, regulatory elements involved in flowering, such as the LFY consensus binding site motif, and numerous RAV1-A binding site motifs involved in leaf maturation and senescence. The presence of these motifs in the *DES1* promoter corroborates the transcriptomic data from *des1* null mutants, which suggest DES1 plays an important role in mature plants.

The guard cell-specific expression of GFP suggests that DES1 is regulated by ABA, and this finding has been recently demonstrated when the stomatal closure in *des1* null mutants was analyzed (Scuffi et al., 2014). Wild type plants closed the stomata in response to exogenous ABA, and *des1* mutants were unable to close the stomata. This lack of response to ABA in *des1* mutants was restored by genetic complementation or by the exogenous application of sulfide. Taken together, these data indicate that DES1 is required for ABA-dependent stomatal closure. It has been demonstrated that DES1 is regulated by ABA at the transcriptional level, specifically in guard cells (Scuffi et al., 2014). Interestingly, it was also observed that the OAS-A1 regulation in response to salt stress is mediated by ABA (Barroso et al., 1999).

GFP expression driven by the *DES1* promoter localizes to sites of high auxin concentration, such as hydathodes (Teale et al., 2006; Wang et al., 2011). This suggests that DES1 may be regulated by auxin, however, we do not observe any root phenotype in the *des1* mutants. At the transcriptional level, we have observed clear repression in the *DES1* transcript level in response to the exogenous application of auxins; the opposite behavior was observed with the *OAS-A1* transcript. The GFP accumulation pattern seems contradictory with the down regulation of *DES1* gene expression by auxin. However, the mechanisms controlling auxin action are very complex and involve auxin biosynthesis, conjugation, catabolism, and transport. At present, we are unable to decipher the specific aspect where DES1 is involved, although we suggest a crosstalk between DES1 and the auxin-signaling pathway.

Auxin regulates a variety of physiological and developmental processes in plants, including senescence. However, different lines of evidence suggest that auxin delays senescence (Lim et al., 2010; Kim et al., 2011) and other evidence suggest that auxin promotes senescence (Hou et al., 2013). Regardless, the premature leaf senescence phenotype observed in the *des1* mutants and also the transcriptional profile that shows the altered expression of auxin-responsive and small auxin up-regulated (SAUR) genes suggest that DES1 is regulated by auxin (Gene Expression Omnibus repository GSE32566) (Alvarez et al., 2012a).

The regulatory relationship between sulfur signaling and auxins was previously demonstrated by analyzing the transcriptional responses of plants to sulfur deficiency. The genes involved in the auxin biosynthesis pathway are up-regulated under sulfur deficiency; this suggests that auxin is involved in the sulfur starvation response (Hirai et al., 2003; Nikiforova et al., 2003). Different sulfur starvation response factors related to auxin signaling have been analyzed, and it was concluded that auxin-related transcriptional regulators coordinate the metabolic shifts induced by sulfur starvation (Falkenberg et al., 2008).

### Conflict of interest statement

The authors declare that the research was conducted in the absence of any commercial or financial relationships that could be construed as a potential conflict of interest.
