# Supplementary figures and images for: Assessing the transcriptional regulation of L-cysteine desulfhydrase 1 in Arabidopsis thaliana
Source: Front Plant Sci. 2014 Dec 4;5:683. doi: 10.3389/fpls.2014.00683 (PMC4255504; doi:10.3389/fpls.2014.00683)

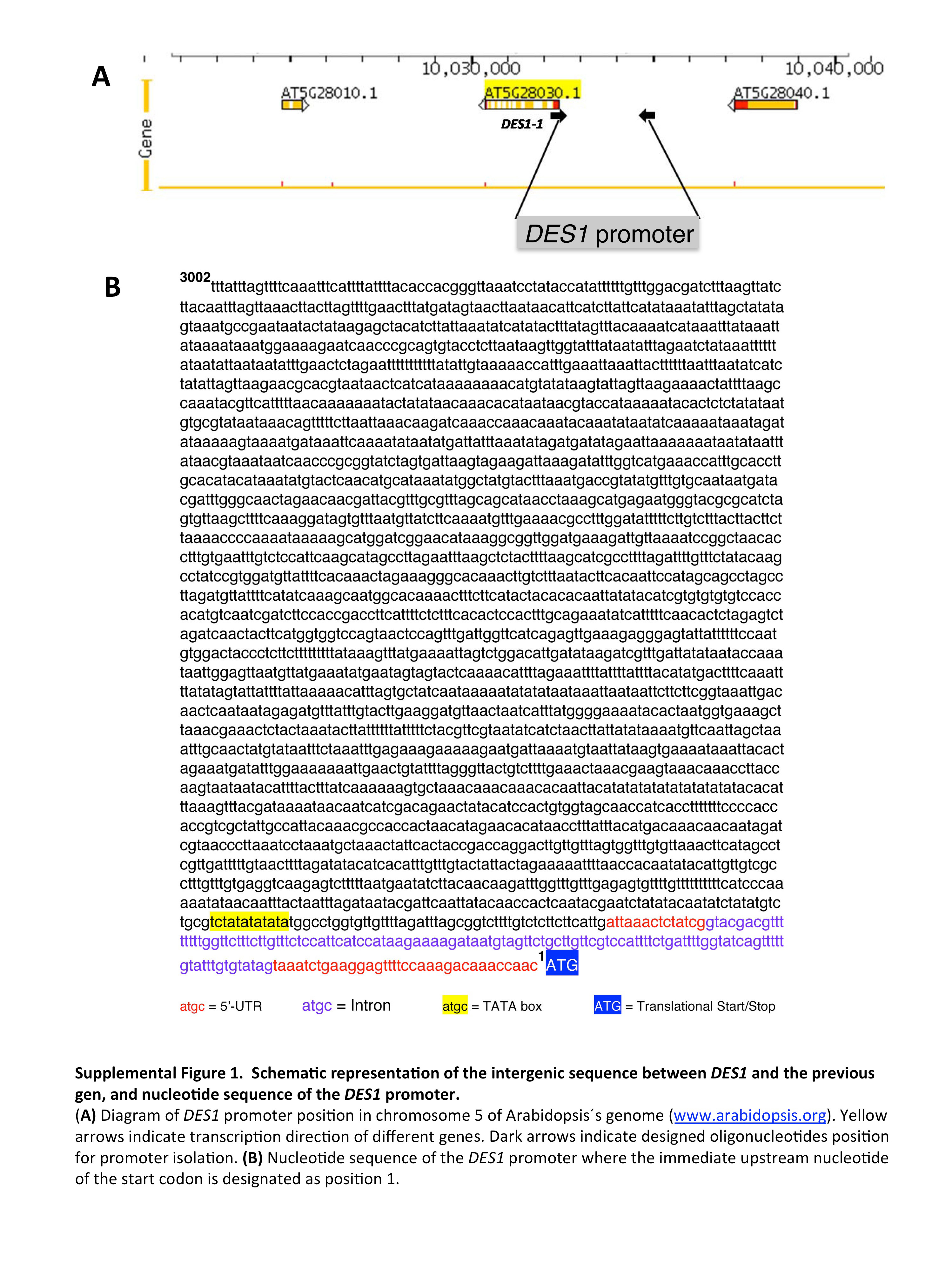

Supplement: Supplementary file 1 [file Image1.JPEG]
